# Nurses’ professional competency and organizational commitment: Is it important for human resource management?

**DOI:** 10.1371/journal.pone.0187863

**Published:** 2017-11-08

**Authors:** Abbas Karami, Jamileh Farokhzadian, Golnaz Foroughameri

**Affiliations:** 1 Nursing Research Center, Kerman University of Medical Sciences, Kerman, Iran; 2 Department of Angiography, Ali-ibn Abi Talib Hospital, Rafsanjan University of Medical Sciences, Rafsanjan, Iran; 3 Department of Community Health Nursing, School of Nursing and Midwifery, Kerman University of Medical Sciences, Kerman, Iran; Cardiff University, UNITED KINGDOM

## Abstract

**Background:**

Professional competency is a fundamental concept in nursing, which has a direct relationship with quality improvement of patient care and public health. Organizational commitment as a kind of affective attachment or sense of loyalty to the organization is an effective factor for professional competency.

**Objective:**

This study was conducted to evaluate the nurses´ professional competency and their organizational commitment as well as the relationship between these two concepts.

**Methods and materials:**

This descriptive-analytic study was conducted at the hospitals affiliated with a University of Medical Sciences, in the southeast of Iran in 2016. The sample included 230 nurses who were selected using stratified random sampling. Data were gathered by three questionnaires including socio-demographic information, competency inventory for registered nurse (CIRN) and Allen Meyer's organizational commitment.

**Results:**

Results showed that professional competency (Mean±SD: 2.82±0.53, range: 1.56–4.00) and organizational commitment (Mean±SD: 72.80±4.95, range: 58–81) of the nurses were at moderate levels. There was no statistically significant correlation between professional competency and organizational commitment (ρ = 0.02; p = 0.74). There were significant differences in professional competency based on marital status (p = 0.03) and work experience (p<0.001).

**Conclusion:**

The results highlighted that the nurses needed to be more competent and committed to their organizations. Developing professional competency and organizational commitment is vital, but not easy. This study suggests that human resource managers should pursue appropriate strategies to enhance the professional competency and organizational commitment of their nursing staff. It is necessary to conduct more comprehensive studies for exploring the status and gaps in the human resource management of healthcare in different cultures and contexts.

## Introduction

In today’s modern world, scholars put an emphasis on the importance and role of human resource in the development of countries; moreover, they believe that the most important capital of each organization is its human resource. In this respect, nurses are taken into account as the biggest and the most important human resource in healthcare organizations [1]. Drastic changes in science and technology, cost containment and insufficient time to establish relationships with patients can lead to increase in the levels of concerns in nurses about patient safety and security, quality of care, nurse safety and security, as well as nurse competency [2,3]. Additionally, modern views to the principles of professionalism emphasize that quality improvement in the health care system is the ethical and professional responsibility of all the medical professions, particularly nurses. Therefore, they should entail commitment to professional competency, honesty with patients and the improvement of the care quality [4].

Professional competency has been proposed as a fundamental element in the provision of nursing care. It should be also noted that professional competency refers to the delivery of nursing care on the basis of professional standards [2]. Nursing competency has been extensively addressed in the literature in terms of safety and quality of nursing care [5]. In fact, professional competency in nurses is defined as a combination of skills, knowledge, attitudes, values and abilities that bring about effective or high performance in occupational and professional positions [6]. Furthermore, professional competency is considered as correct judgment and habits in terms of the use of knowledge, technical skills, clinical reasoning, communication, feelings, values and rethinking daily activities aimed at providing services to individuals and the society [7].

Professional empowerment and competency of nurses are among the concerns of human resource management in healthcare systems worldwide. World Health Organization (WHO) requires all the member countries to report and implement their plans for strengthening nurses and equipping them with professional competency [8]. Having competency leads to an improved quality of patient care and an increased patient satisfaction with the nurses and helps promote nursing as a profession and improve nursing education and clinical nursing [9]. In addition, patients expect nurses to be competent and to behave them in a reasonable way. Following high prevalence of medical incidents, the government, the media, and the public have become concerned about the quality of clinical care and have focused their attentions on clinicians’ competency. There is a need for professionals to demonstrate that they are clinically competent to perform certain roles [10]. In this respect, lack of attention to professional competency in nurses can cause problems for organizations and question their activities. Nurses' poor competency may lead to some undesirable consequences including nurses' frustration, job dissatisfaction, and their attrition [5].

Professional skills and competency also have effects on job attitudes including organizational commitment and professional affiliations [11]. In order to achieve the goals of the health system, manpower is required to have not only expertise, empowerment and competency, but also high levels of organizational attachment and commitment as well as willingness to become involved in the activities beyond their common and pre-determined duties. Therefore, the levels of attachment and commitment of nurses towards their affiliated organizations can have impacts on the promotion of their clinical competency [12]. In this respect, organizational commitment is defined as involvement in a particular organization and beliefs in values and goals of the organization, sense of loyalty to the organization, moral obligations, heartfelt inclinations and sense of need to stay in the organization [13]. In fact, organizational commitment is a type of psychological attachment to an organization in which a person is involved so that committed employees sometimes obtain their own identity from the organization and benefit from their memberships [14]. Organizational commitment is also considered as one of the basic values, which affects an organization, and it is used as a criterion to evaluate employees [15].

Consequences of organizational commitment include lower levels of intent to leave, increased retention, better attendance, and higher job productivity [13]. Han& Chung [16] have highlighted that nurses’ organizational commitment is an essential precondition not only for the reduction of negative consequences such as conflicts, exhaustion and turnover, but also for the maintenance of patients’ health through a deeper commitment to patients. Members who are highly committed to their organization tend to earn external as well as internal remuneration (e.g., job satisfaction), to maintain friendly relationships with coworkers, and to perform tasks in favor of the organization.

Lower levels of organizational commitment or its shortage can likewise lead to a series of problems in an organization including turnover, absenteeism, the decreased quality of health care, inconsistencies with organizational goals, declines in organizational earnings and loads of other difficulties [15]. Nurses and organizations are two inseparable factors affecting each other in the field of health; however, the results of nursing activities can be satisfied when they meet their organizational commitment, have professional skills and competency and know themselves as a part of an organization they are involved in. Such individuals prefer organizational goals to personal and ethnic ones and always take organizational excellence into account [17].

The review of the related literature shows that professional growth has an increasingly positive effect on organizational commitment. For example, Weng et al., [18] in a large study in China reported that all four dimensions of professional growth of employees (career goal progress, professional ability development, promotion speed and remuneration growth) positively were correlated with organizational commitment. Results of a study in the USA revealed that nurses’ professional empowerment is associated with the organizational commitment and mediates effects of organizational conflict and trust on the commitment to the organization [13]. In another study, the organizational commitment has decreased levels of work-related stress [16]. In a study by Ingersoll et al., [19] job satisfaction, the organizational commitment, professionalism and professional performance were assessed in nurses. Researchers also reported relationships between organizational and personal factors and job satisfaction. Most individuals with higher job satisfaction also paid attention to their professional empowerment. Therefore, educational efforts, the organizational commitment and programs for nurses to stay in their jobs were proposed in this study.

With this regard, a few studies have been conducted in Iran; for example, Niazazari et al., [20] argued that professional ethics was positively correlated with the organizational commitment. In addition, it was concluded that the professional ethics as a dimension of professional competency had the power to predict the organizational commitment of employees. Another study reported that the nurses were deprived of appropriate professional competency to provide spiritual care [21].

Given the above-mentioned issues, it can be argued that the professional competency and the organizational commitment can both have effects on the employee’s quality of work and life in each organization and, finally, the quality of nursing care. Furthermore, the researchers of this study with work experience as clinical nurses or nursing managers at hospitals as well as clinical instructors emphasized the evaluation of the professional competency and the organizational commitment as well as their inter-relationships. They had observed problems and the effects of these two important features on providing patient care and treatment. Additionally, the professional competency and the organizational commitment are linked with organizational context, such as hospital status and size, and the status of human resource management. Given the scarcity of the related studies in the culture and context of Iranian hospitals, the present study was conducted to evaluate: (1) the nurses’ professional competency, (2) their organizational commitment and (3) the relationship between the professional competency and the organizational commitment.

The results of this study can help managers in terms of policy-making to provide the infrastructure of appropriate management of human resource and plan for the dimensions of the professional competency and the organizational commitment of nurses in both developed and developing countries.

## Methods

### Study design and settings

This descriptive-analytic study was conducted at two teaching and referral hospitals affiliated with Rafsanjan University of Medical Sciences (RUMS) in the southeast of Iran from March to June 2016. At the time of data collection, these hospitals were small and medium in size with 100 and 300 beds, respectively.

### Participants and sampling

The target population of this study included nurses working at the time of data collection (N = 364). The inclusion criteria were having Bachelor’s degrees or higher, work experience over 6 months, working in the position of a clinical nurse or a head nurse at the time of data collection and good mental and psychological conditions. Incomplete questionnaires were considered as the exclusion criteria. The sample size was calculated based on Cochran’s formula (α = 0.05, d = 0.05, Z = 1.96) by 190 individuals and, in total, 240 nurses were recruited in the study by taking into account the probability of participant loss. The studied samples were selected through stratified random sampling based on the proportion of nurses working per hospital. Finally, 10 questionnaires were excluded from the study due to their incompleteness (the response rate was 95.83%).

### Instruments and data collection

Three questionnaires were used for data collection. The first part was a demographic questionnaire including items about age, gender, marital status, work shifts, level of education, type of employment, position and work experience.

The second part included competency inventory for registered nurse (CIRN) that was introduced by Liu et al., (2009). This questionnaire evaluates the competency of nurses in various clinical positions in the form of self-evaluation or peer evaluation [22]. The Persian version of the questionnaire was also translated and validated in 2014 by Ghasemi et al., [23]. This questionnaire consisted of 55 items and 7 dimensions including clinical care (10 items), leadership (9 items), interpersonal relationships (8 items), legal-ethical practice (8 items), professional development (6 items), teaching-coaching (6 items) and research aptitude-critical thinking (8 items).

The CIRN was scored using the five-point likert scale ranging from 0 to 4, with incompetency (score zero), low competency (score 1), moderate competency (score 2), high or sufficient competency (score 3) and very high competency (score 4). In this respect, the mean scores below 2, 2–3 and above 3 were considered as low competent, moderate and highly competent, respectively.

The content validity index of the main questionnaire calculated by experts was 85%. In order to determine the reliability of the questionnaire, Cronbach’s alpha coefficient was used in which Cronbach’s alpha of the entire instrument was 0.90, and it was reported in the range of 71–90% for its dimensions [22]. The Content Validity Index (CVI) of the Persian version of CIRN was 94% for the entire instrument and more than 83% for all the items. Furthermore, the reliability of the questionnaire was calculated through internal consistency method, in which Cronbach’s alpha coefficient for the whole instrument was 0.97 and it was reported between 68–87% for all the dimensions of this inventory [23].

The third part was the organizational commitment questionnaire which was developed in 1990 by Allen and Meyer. Bastami et al., [15] used the Persian version of this questionnaire in 2013. This questionnaire was comprised of 24 items, in which each of the 8 items measured one dimension of the organizational commitment, i.e. affective commitment, normative commitment and continuance commitment. Responses to this questionnaire were based on a 5-point Likert scale, with strongly agree (score 5), agree (score 4), neutral (score 3), disagree (score 2) and strongly disagree (score 1). The minimum score in this questionnaire was 24 and the maximum score was 120. The scores between 90 and 120 were considered as high organizational commitment, scores between 60–90 and lower than 60 were likewise taken into account as moderate and low organizational commitment, respectively. In general, higher scores indicated the greater organizational commitment of an individual.

### Ethical considerations

The Ethics Committee affiliated with Kerman University of Medical Sciences approved the conductance of this study as well as the consent procedure (Medical Ethic No: ir.kmu.rec.05.1395). Thus, first, the researcher presented the letter of introduction for the required coordination with the context of the study. A cover letter explaining the purpose of the study and the procedure for the data collection was provided to the eligible participants prior to the data collection. Then verbal agreement of the participants was obtained, and they were ensured in terms of confidentiality and anonymity of the data as well as voluntary participation in the study. Informed consent was implied from returning the completed questionnaires.

### Statistical analysis

Descriptive statistics (percentage, mean and standard deviation) as well as analytical statistics (Mann-Whitney test, Kruskal-Wallis test and Spearman correlation coefficient) were used. The level of significance was considered by 5% and SPSS software (version 18) was employed for data analysis.

## Results

### Demographic characteristics

The results showed that the majority of the respondents were female (65.7%) and married (79.1%). In terms of the level of education, 93.5% had Bachelor’s degrees; they also had 6 to 15 years of work experience (46.1%). About 94% of the respondents were employed in the position of a regular nurse and their age (53.9%) was between 30 and 40 years old. The employment status of 52.7% of the nurses was permanent and about 82.1% of them also worked in rotating shifts (Table 1).

**Table 1:** Nurses’ demographic information and its relationship with professional competency and organizational commitment mean scores (n = 230).

Variables	categories	n(%)	mean	p-Value	mean	p-Value
			Professional competency		organizational commitment	
Gender	Female	151 (65.7)	2.80	0.59	70.76	0.60
	Male	79 (34.3)	2.84		71.02	
Marital status	Married	182 (79.1)	2.87	0.03	70.67	0.32
	Single	48 (24.4)	2.68		71.54	
Age (years)	<30	77 (33.5)	2.77	0.22	69.8	0.08
	30–40	124 (53.9)	2.84		71.23	
	>40	29 (12.6)	2.94		72.00	
Level of education	Bachelor	215 (93.5)	2.82	0.23	70.81	0.69
	Master’s & higher	15 (6.5)	2.99		71.40	
Position	Head nurse	14 (6)	3.12	0.07	68.71	0.06
	Nurse	216 (94)	2.81		70.99	
work of experience (years)	up to 5	91 (39.6)	2.64	p<0.001	71.19	0.19
	6–15	106 (46.1)	2.94		70.20	
	>15	33 (14.3)	2.97		72.03	
Type of employment	Hired	121 (52.7)	2.87	0.10	70.57	0.11
	fixed-time ^a^	79 (34.3)	2.83		71.76	
	Contract ^b^	30 (13)	2.6		68.57	
Shift work	Fixed	41 (17.9)	2.99	0.06	70.05	0.12
	In rotation	189 (82.1)	2.79		71.03	

Notes

^a^ annually contracted with payment similar to hired nurses

^b^ annually contracted with payment less than hired nurses.

### Professional competency

The results of the evaluation of professional competency demonstrated that 58.3% of the nurses had moderate professional competency and 34.3% achieved high competence professionally. Table 2 shows that the mean score of professional competency of the nurses was at the moderate level (2.82±0.64, range: 1.56–4.00). The highest mean score was also related to “legal/ethical practice” (3.13±0.60, range: 1.13–4.00) and the lowest mean score was associated to “research aptitude/creative thinking” (2.70±0.67, range: 0.88–4.00).

**Table 2:** Mean score of nurses’ professional competency.

Dimensions	Minimum	Maximum	Mean	SD
Clinical care	1.20	4.00	2.85	0.58
Leadership	1.33	4.00	2.78	0.59
Interpersonal relationships	1.00	4.00	2.77	0.61
Legal/ethical practice	1.13	4.00	3.13	0.60
Professional development	0.67	4.00	2.78	0.65
Teaching–coaching	0.00	4.00	2.76	0.78
Research aptitude/ Critical thinking	0.88	4.00	2.70	0.67
Total	1.56	4.00	2.82	0.53

The results of the Mann-Whitney U test indicated a significant difference between the mean scores of the professional competency based on marital status so that the level of professional competency in married individuals was higher than that of the unmarried (P = 0.03, Z = -2.15). Moreover, the results of Kruskal-Wallis test showed a significant difference in professional competency based on work experience groups so that the nurses with the work experience over 15 years had higher professional competency than others (χ2 = 18.2, df = 2, P<0.001). There were no significant differences in professional competency based on other demographic characteristics (P˃0.05) (Table 1).

### Organizational commitment

All the nurses in this study (100%) had moderate levels of the organizational commitment and the overall mean score for their commitment was (72.80±4.95, range: 58–81). The highest and the lowest mean scores of the organizational commitment were related to “affective commitment and continuous commitment”, respectively (Table 3). No significant differences were found between the organizational commitment and the demographic characteristics (P˃0.05) (Table 1).

**Table 3:** Mean score of nurses’ organizational commitment.

Dimensions	Minimum	Maximum	Mean	SD
Affective Commitment	12	33	24.49	3.55
Continuance Commitment	16	34	23.93	3.43
Normative Commitment	10	33	24.38	3.71
Total	58	81	72.80	4.95

### Correlation between professional competency and organizational commitment

The results of Spearman’s coefficient revealed no statistically significant correlation between the professional competency in the nurses and their organizational commitment (ρ = 0.02, P = 0.74). Therefore, the increasing scores of the professional competency did not have any effect on the increased scores of organizational commitment. However, there was a significant, but poor correlation between the normative commitment and the dimensions of interpersonal relationships (ρ = 0.13, P = 0.04) as well as professional development (ρ = 0.18, P = 0.006). Besides, the affective commitment had statistically poor and direct correlation with the clinical care (ρ = 0.18, P = 0.007). There was likewise no significant correlation between other dimensions of the professional competency and the organizational commitment (Table 4).

**Table 4:** Correlation between nurses’ professional competency and organizational commitment.

Variable	Affective Commitment	Continuance Commitment	Normative Commitment	Total Organizational Commitment
Clinical care	ρ = 0.18 P = 0.007	ρ = -0.03 P = 0.58	ρ = 0.04 P = 0.5	ρ = 0.00 P = 0.93
Leadership	ρ = 0.01 P = 0.84	ρ = -0.04 P = 0.52	ρ = 0.08 P = 0.23	ρ = -0.01 P = 0.85
Interpersonal relationships	ρ = -0.08 P = 0.19	ρ = 0.009 P = 0.89	ρ = 0. 13 P = 0.04	ρ = -0.002 P = 0.97
Legal/ethical practice	ρ = 0.11 P = 0.08	ρ = -0.04 P = 0.53	ρ = 0.06 P = 0.31	ρ = 0.01 P = 0.83
Professional development	ρ = 0.07 P = 0.27	ρ = -0.02 P = 0.66	ρ = 0.18 P = 0.006	ρ = 0.09 P = 0.15
Teaching–coaching	ρ = 0.01 P = 0.82	ρ = -0.003 P = 0.96	ρ = 0.05 P = 0.4	ρ = -0.02 P = 0.68
Research aptitude/ Critical thinking	ρ = 0.06 P = 0.36	ρ = -0.05 P = 0.38	ρ = 0.05 P = 0.41	ρ = -0.01 P = 0.89
Total Professional competency	ρ = 0.06 P = 0.32	ρ = -0.03 P = 0.59	ρ = 0.10 P = 0.1	ρ = 0.02 P = 0.74

## Discussion

### Principal findings

The present study was aimed to evaluate the nurses´ professional competency and their organizational commitment as well as the relationship between these two concepts. The results showed that the professional competency and the organizational commitment of the nurses were at moderate levels and no statistically significant relationship was found between the organizational commitment and the professional competency. In line with this study, researchers in Iran and other countries have also examined the competency of nurses through self-evaluation methods and the results of their studies have been moderate [24–26]. In contrast with this study, in several studies, the majority of nurses assessed their overall nursing competencies as good and very good using self-evaluation [4,5,26–28]. Two studies reported that newly graduated nurses were weak in terms of their clinical competency [29,30]. In another study, it was revealed that nurses did not have appropriate professional competency to provide spiritual care, and they received no training in this respect. The results of the study showed the necessity to conduct further investigations in the fields of the professional competency and the spiritual care [21]. The difference in the results of the present study and the cited studies could be associated with the discrepancy in the samples and relevant measurement instruments for professional competency. Moreover, it should be noted that professional competency is a multi-faceted phenomenon, which can be related to nursing education system, views and attitudes of the system to nurses and nursing and socio-economic as well as cultural factors. Lack of sufficient motivation in nurses, occupational burnout, low quality and quantity of educational courses, lack of job satisfaction and professional interest, disproportionate recruitment of nursing staff in terms of number of patients and lack of clear standards for professional competency are the factors that may cause reduced levels of clinical competency in nurses. The results of a qualitative study showed that factors such as experience, opportunities, environment, personal characteristics, motivation and theoretical knowledge were among the most important factors affecting clinical competency of nurses [31].

Moreover, the results of the present study demonstrated that the highest mean score of the professional competency was related to “the legal/ethical practice” dimension and the lowest score was associated with “research aptitude and critical thinking”. This result is in agreement with studies by Liu et al., [27] and Karimi-Moonaghi et al., [32]. One of the reasons for the high levels of the professional competency in the legal/ethical practice dimension could be explained by the nature of nursing work that nursing is a profession with inherently legal/ethical practice and these are among the professional values which are frequently highlighted in nursing educational courses and at the work place. Furthermore, one of the reasons with this regard can be the approaches of quality improvement such as clinical governance and accreditation, which have been used in healthcare organizations. Legal and ethical issues play substantial roles in these new approaches. However, the present study result was not compatible with that of Bahreini et al., [33] which reported the maximum score in “management” domain. The differences between scores can be due to different evaluation methods, various points of views and expectations of the individuals of their own roles [32]. In another study, Intensive Care Unit (ICU) nurses rated the domain of “ethical activity and familiarity with healthcare laws” as low. This incompatibility can be explained by the fact that ICU nurses at bed-side nursing care are more familiar with safe, direct patient- centered care (when using these competencies frequently in daily care) than with adherence to ethical codes, general health care legislation, and transplantation legislation [28]. Salonen et al., [26] reported that overall competency and the level of competency in different categories varied between different types of units. Competency profiles therefore are context-specific and differ between different practice settings.

In the present study, the lowest mean score for the dimensions of professional competency was related to “research aptitude and critical thinking”. In line with this study, Squires et al., [34] argued that nursing care was still tailored to tradition and the nurses were not willing to undertake research in their area of expertise and failed to use the research findings. In another study, one of the least important professional values based on the nurses’ perspective, was “Participating in nursing research and/or implementing research findings appropriate to practice [35]. The reasons for lower research-related competency are probably the inappropriate attitudes towards the use of research- and evidence-based practice and low self-efficacy in this field as well as weaknesses in information literacy skills such as searching for relevant information resource, manner of data organization in databases, skills for data recovery and evaluation of evidence. These factors were emphasized in two studies [36,37]. The study by Conner [38] can be also assumed as the one inconsistent with the results of the present study. Researcher found that a large number of nurses considered research-based practice and only a few of them took this approach into account as waste of time. Therefore, continuous education was recommended to strengthen such competencies of the nurses. In this respect, the duties associated with research activities related to professions including attendance in organizational meetings, participation in research studies and application of their results in practice can strengthen the professional competency at the work place. According to Farokhzadian et al., [3] nursing managers are responsible for transforming nurses’ work environment and promoting safety and quality care in clinical environments. The leadership behaviors of nursing managers are important for changing clinical settings. They should be role models and must be able to influence the promotion of research-related behaviors among nurses.

The results of this study also indicated that the mean score of professional competency of married nurses was higher than that of the single. Han & Chung [16] reported that insufficient professional knowledge and skills were affected by marital status. In contrast to the results of the present study, the findings of two studies [21,39] showed that the clinical competency was not significantly associated with marital status. Probably, it can be said that professional promotion and nursing competency are affected by the individuals’ personal and socio-economic as well as cultural factors.

In this study, the professional competency in the nurses over15-year work experience was greater than that of other groups. The results are similar to the previous studies in Iran and other countries that showed a difference between the levels of competency based on work experience [21,26,39]. It can be assumed that more experienced employees could better adapt themselves to different situations with high sense of competency and, consequently, they are more empowered due to utilizing their past experiences.

Furthermore, the findings of this study indicated that the level of organizational commitment was at the moderate level. Previous studies in the U.S. [40], South Korea [41], Canada [42] and in Iran [43,44] were also in agreement with the present study in terms of their reports on the moderate levels of the organizational commitment of nurses. In another study, participants were found to have a moderate level of commitment to their employers. Among the factors that significantly affected commitment were the participants’ educational level, perceived level of organizational support, role clarity and organizational leadership [45].

In the present study, no nurses were observed with high organizational commitment, which was thought-provoking and in conflict with the studies by Ahamd & Oranye [14] and Lorber & Skela-Savic [46] who reported that the majority of participants were endowed with a high level of the organizational commitment. Researchers in a study highlighted that organizational climate indirectly affected the organizational commitment and intention to leave. A positive organizational climate can increase the nurses’ commitment to their organization and reduce their intention to leave. Creation of an excellent organizational climate as a fundamental orientation towards the organization’s missions and strategic goals increases employees’ satisfaction and brings about better conditions for the nurses’ interactions and reduces intention to leave among them [47]. Sikorska-Simmons [48] found that an empowering work environment and organizational culture, job satisfaction, and levels of education were the strongest predictors of the commitment. Researcher concluded that the organizational culture, value and the respect of staff members were the most effective on promoting higher levels of the organizational commitment. Therefore, strengthening and increasing the organizational commitment of nurses seem necessary and vital.

In this study, the highest mean score belonged to “affective commitment”. It is argued that the employees who earn high scores in the given commitment tend to remain in an organization and are less willing to leave there in order to continue working in other organizations. Moreover, the lowest mean score in this study was related to “continuance commitment”. In Iran, these results were consistent with the findings of a study [49], and also disagreed with another study [44]. It should be noted that the continuance commitment is associated with the benefits and costs of staying in/leaving an organization. Ahmad & Oranye [14] explained that affective commitment appeared to have a more significant influence on job satisfaction among nurses compared with the other two components of the organizational commitment. This agrees with a generally accepted view that the affective commitment has the strongest and most consistent relationship with desirable outcomes. The nursing profession is essentially about care and affection is crucial.

The results also showed no statistically significant correlation between the professional competency of the nurses and their organizational commitment. In contrast with present study, several studies in other countries and Iran revealed that a significantly positive correlation was reported between the professional competency and the organizational commitment [13,18,50]. However, the normative commitment was significantly and directly correlated with the dimensions of interpersonal relationships and professional development, but in a poor manner. Moreover, there was a significant and direct, but poor, relationship between the normative commitment and the dimensions of clinical care. In line with the present study, studies have reported significant relationships between the empowerment and the organizational commitment of nurses [51], the professional growth and the organizational commitment [18] as well as the psychological empowerment and the organizational commitment [52]. Also a systematic review showed that all three forms of the commitment related negatively to withdrawal cognition and turnover, and the affective commitment had the strongest and most favorable correlations with the organization-relevant (attendance, performance, and organizational citizenship behavior) and employee-relevant (stress and work–family conflict) outcomes [53]. Weng et al., [18] highlighted that when the organization provides a good career growth platform for their employees by helping them meet career goals and enhance their professional abilities, and rewards them in return via promotions and remuneration, those employees are more apt to reciprocate and develop a sense of moral obligation toward the organization.

No significant correlation between the organizational commitment and the professional competency can be investigated from various aspects. In the present study, there was no high level of the organizational commitment and all were moderate indicating that lack of high organizational commitment emanates from macro-management causes and depends on the views and attitudes of nurses to their organizations. Factors such as fulfillment of duties, accountability, love and tendency to work, dedication, effectiveness and dynamicity as well as work condition, its importance, work autonomy and organization conditions can affect the organizational commitment that consequently has an impact on turnover rate. For example, in a qualitative study by Heidari et al., [54] lack of sense of attachment, confidence and satisfaction with professional position were identified as the main concerns of nurses in the process of organizational learning. Also, the nurses highlighted issues such as lack of confidence at the obtained competencies, deficiency in work autonomy, no decision-making power, absence of autonomy to use their knowledge and predominance of physicians’ opinions which had deterrent effects on the efforts of the nurses to achieve personal and organizational promotion. The interesting point about the experiences of nurses was that, in the current system, higher knowledge and skills not only were considered as benefits to nurses, but also granted more duties and responsibilities to them. In another qualitative study, nurses believed that managers acted very weakly in the empowerment of human resource. Factors such as ineffective educational programs, lack of motivation, inappropriate evaluation of performance, inadequate productivity, inappropriate staffing and financial resource created the challenge of recession of empowerment for human resource management [55]. Additionally, a study suggested that the perceptions of the individuals from the amount of justice in an organization had effects on their attitudes to the organizational commitment [56]. Feeling lack of justice in evaluations along with dissatisfaction can lead to failure in the evaluation system. It can also lead to reduction in employees’ spirit, the organizational commitment and willingness to achieve and promote professional competency and productivity.

### Limitations

This study had two limitations. First, the use of questionnaire to assess the professional competency and the organizational commitment may have resulted in exaggerative scores and could be a subject to personal bias. Future studies can be performed using different methods of competency evaluation such as 360 degree evaluation method or evaluating via new and scientific methods to help determine the actual competency. To increase the reliability of the findings, triangulation in data collection such as interviews and observation can be helpful as well. Second, this study was limited to the nurses in two teaching hospitals affiliated with a medical university in Iran, which may question the generalizability of the results. However, the distribution of nurses and the organization of healthcare institutions are similar in the whole country because of the centralized healthcare policy-making system in Iran [36].

## Conclusions

The results of this study showed that these nurses had no high professional competency and the organizational commitment. There was also no statistically significant relationship between the professional competency and the organizational commitment. It is suggested that nursing managers should distinguish the importance of the organizational commitment and the professional competency for the high-quality and safety in healthcare. The findings also provide a basis for human resource managers thus they pursue more efficient steps to promote the professional competency and the organizational commitment of their nursing staff. In this case, it may be useful to apply strategies such as providing adequate management support for the nurses and allowing them to grow professionally, fair evaluation; sense of autonomy, as well as welfare of employees and improving organizational resource (financial and human resource along with time allocation). This can be accomplished by planning and developing educational courses by nursing managers, policy- and decision-makers, and nursing educators. In addition, we suggest to conduct comprehensive qualitative and quantitative studies for exploring the status and gaps of human resource management in healthcare, and also to evaluate the effect of different approaches to enhance the professional competency and the organizational commitment of healthcare providers in different cultures and contexts.

## Supporting information

S1 File A minimal set of data for the study. (SAV)
